# Life satisfaction in persons with mental disorders

**DOI:** 10.1007/s11136-020-02556-9

**Published:** 2020-06-16

**Authors:** Adrian Meule, Ulrich Voderholzer

**Affiliations:** 1 Department of Psychiatry and Psychotherapy, University Hospital, LMU Munich, Munich, Germany; 2 Schoen Clinic Roseneck, Am Roseneck 6, 83209 Prien am Chiemsee, Germany; 3 Department of Psychiatry and Psychotherapy, University Hospital of Freiburg, Freiburg, Germany

**Keywords:** Satisfaction With Life Scale, Life satisfaction, Measurement invariance, Mental disorders, Inpatient treatment

## Abstract

**Purpose:**

Life satisfaction refers to a cognitive and global evaluation of the quality of one’s life as a whole. The arguably most often used measure of life satisfaction is the Satisfaction With Life Scale (SWLS). Persons with mental disorders generally report lower SWLS scores than healthy controls, yet there is a lack of studies that have compared different diagnostic groups, tested measurement invariance of the SWLS across these groups, and examined effects of treatment on life satisfaction.

**Methods:**

Data of 9649 inpatients of seven diagnostic categories were analyzed: depressive episode, recurrent depressive disorder, phobic disorders, obsessive-compulsive disorder, trauma-related disorders, somatoform disorders, and eating disorders.

**Results:**

The one-factor structure of the SWLS was replicated and full measurement invariance was demonstrated across groups. Patients with trauma-related disorders reported the lowest life satisfaction. Life satisfaction significantly increased during treatment across all groups and these changes were moderately related to changes in depressive symptoms.

**Conclusions:**

Results support the excellent psychometric properties of the SWLS. They also demonstrate that although persons with mental disorder generally report lower life satisfaction than persons without mental disorders, life satisfaction also varies considerably between different diagnostic groups. Finally, results show that life satisfaction increases during inpatient treatment, although at discharge most patients have rarely reached levels of life satisfaction reported in non-clinical samples.

## Introduction

Life satisfaction can be defined as a cognitive and global evaluation of the quality of one’s life as a whole [1]. The Satisfaction With Life Scale (SWLS [2]) is arguably the most often used instrument for measuring life satisfaction. As of January 2020, using the search term “Satisfaction With Life Scale” resulted in more than 43,000 hits in Google Scholar and the article by Diener and colleagues [2] has been cited—according to Google Scholar—more than 25,000 times. The SWLS consists of five items and responses are recorded on a seven-point scale ranging from 1 = *strongly disagree* to 7 = *strongly agree*. Thus, sum scores can range between five and 35 and higher scores represent higher life satisfaction.

Internal reliability of the SWLS has been found to be good (around α = 0.80) across several studies and samples [3]. Similarly, the SWLS has been found to have a one-factor structure, which has been replicated numerous times [1, 4, 5]. Another important aspect of the psychometric properties of a test, however, is measurement invariance, which indicates that the same construct is being measured across different groups or points in time. Although a large amount of studies have examined measurement invariance of the SWLS across sex, age, different countries, or points in time (for overviews see [1, 5, 6]), results have been mixed. For example, while measurement invariance has been fairly well established across men and women, this has rarely been found for different age or cultural groups [7]. Furthermore, measurement invariance has not been tested across other groups, for example, across different groups of persons with mental disorders. However, establishing measurement invariance of the SWLS across these groups is important as it is a prerequisite for comparing scores between these groups. That is, violations of measurement invariance may preclude meaningful interpretations of group differences in SWLS scores.

In unselected or healthy samples, mean sum scores of the SWLS roughly range between 20 and 30, indicating that participants are slightly or largely satisfied with their lives [1, 4]. In persons with physical diseases or other health concerns, scores are usually lower than 20, although there is considerable variation between such groups [1, 4]. To date, the lowest scores—thus, the lowest life satisfaction—have been reported in individuals with traumatic brain injury and post-traumatic stress disorder, in male prison inmates and in sex workers, with mean sum scores of about 13, 12, and 10, respectively [1, 4].

Studies that examined persons with mental disorders have largely reported lower SWLS scores than in participants without mental disorders. For example, lower scores have been reported in heterogeneous samples of persons with mental disorders [8, 9], in obese women with binge eating disorder [10], or in persons with obsessive–compulsive disorder [11] than in healthy control participants. In a study that compared several diagnostic groups, lower SWLS scores were found in all groups except persons with hypomania and bipolar disorder compared to persons without mental disorders [12].

As persons with mental disorders report reduced life satisfaction, the question arises whether and to which extent life satisfaction can be improved. While evidence-based treatments lead to symptom reductions, “clinical practice should not just endeavor to alleviate misery, but should also strive to build rewarding lives” ( [1], p. 146). While previous studies suggest that scores on the SWLS have moderate temporal stability, they are also subject to change over time [1, 4]. Indeed, preliminary evidence from a study in 25 patients suggests that life satisfaction as measured with the SWLS increases during psychotherapeutic treatment [13].

The current study examined life satisfaction as measured with the SWLS in different diagnostic groups of persons with mental disorders who received inpatient treatment. A first aim was to examine the factor structure of the SWLS and to test measurement invariance across the different diagnostic groups. A second aim was to examine group differences in SWLS scores as well as changes over time (that is, from admission to discharge). A third aim was to explore whether such effects related to sex, age, and length of stay at the hospital. Finally, as the SWLS negatively correlates with affective aspects of subjective well-being such as depression [1, 4], we also examined whether group differences and changes in life satisfaction parallel those in depressive symptoms or whether they are partially independent from depressive symptoms.

## Methods

### Sample

Clinical records of inpatients treated at the Schoen Clinic Roseneck (Prien am Chiemsee, Germany) between 2014 and 2019 were analyzed. The German version of the SWLS [5] and the depression scale of the Patient Health Questionnaire (PHQ–9 [14–16]) are part of the routine diagnostic assessment at the hospital and are completed by the patients both at admission and at discharge. Only patients without missing SWLS data at admission and discharge were included in the current analyses. Moreover, only data from diagnostic groups with *n* > 300 were included as smaller group sizes are generally considered as not appropriate for confirmatory factor analysis ([17]; the largest group of patients that was excluded because of this procedure was other anxiety disorders [F41; *n* = 164]). The final sample with complete SWLS data at both admission and discharge was *N* = 9649 patients (*n* = 9610 for PHQ–9 data) and included patients of seven diagnostic categories (based on ICD–10 classification): depressive episode, recurrent depressive disorder, phobic disorders, obsessive–compulsive disorder, trauma-related disorders, somatoform disorders, and eating disorders (Table 1).

**Table 1:** Sample characteristics

*N* = 9649	Depressive episode (F32)	Recurrent depressive disorder (F33)	Phobic disorders (F40)	Obsessive–compulsive disorder (F42)	Trauma-related disorders (F43)	Somatoform disorders (F45)	Eating disorders (F50)
Group size	*n* = 1946	*n* = 2697	*n* = 465	*n* = 909	*n* = 538	*n* = 340	*n* = 2754
Sex (female)	*n* = 1163 (59.8%)	*n* = 1638 (60.7%)	*n* = 258 (55.5%)	*n* = 548 (60.3%)	*n* = 461 (85.7%)	*n* = 234 (68.8%)	*n* = 2653 (96.3%)
Age (years)	*M* = 41.6 (*SD* = 16.6)	*M* = 46.6 (*SD* = 14.6)	*M* = 30.2 (*SD* = 14.5)	*M* = 32.9 (*SD* = 13.7)	*M* = 41.0 (*SD* = 13.7)	*M* = 45.2 (*SD* = 16.0)	*M* = 23.5 (*SD* = 10.5)
Length of stay (days)	*M* = 54.6 (*SD* = 24.5)	*M* = 53.0 (*SD* = 18.9)	*M* = 60.7 (*SD* = 21.6)	*M* = 71.7 (*SD* = 24.0)	*M* = 71.5 (*SD* = 23.8)	*M* = 49.2 (*SD* = 17.8)	*M* = 93.5 (*SD* = 43.2)
Specific diagnoses	Mild depressive episode (F32.0, *n* = 4, 0.2%) Moderate depressive episode (F32.1, *n* = 1257, 64.6%) Severe depressive episode without psychotic symptoms (F32.2, *n* = 677, 34.8%) Severe depressive episode with psychotic symptoms (F32.3, *n* = 6, 0.3%) Depressive episode, unspecified (F32.9, *n* = 2, 0.1%)	Recurrent depressive disorder, current episode mild (F33.0, *n* = 4, 0.1%) Recurrent depressive disorder, current episode moderate (F33.1, *n* = 1426, 52.9%) Recurrent depressive disorder, current episode severe without psychotic symptoms (F33.2, *n* = 1253, 46.5%) Recurrent depressive disorder, current episode severe with psychotic symptoms (F33.3, *n* = 12, 0.4%) Other recurrent depressive disorders (F33.8, *n* = 1, 0.04%) Recurrent depressive disorder, unspecified (F33.9, *n* = 1, 0.04%)	Agoraphobia (F40.0, *n* = 183, 39.4%) Social phobias (F40.1, *n* = 248, 53.3%) Specific phobias (F40.2, *n* = 30, 6.5%) Other phobic anxiety disorders (F40.8, *n* = 3, 0.6%) Phobic anxiety disorder, unspecified (F40.9, *n* = 1, 0.2%)	Predominantly obsessional thoughts or ruminations (F42.0, *n* = 51, 5.6%) Predominantly compulsive acts (F42.1, *n* = 171, 18.8%) Mixed obsessional thoughts and acts (F42.2, *n* = 683, 75.1%) Obsessive–compulsive disorder, unspecified (F42.9, *n* = 4, 0.4%)	Post-traumatic stress disorder (F43.1, *n* = 531, 98.7%) Other reactions to severe stress (F43.8, *n* = 7, 1.3%)	Somatization disorder (F45.0, *n* = 58, 17.1%) Undifferentiated somatoform disorder (F45.1, *n* = 41, 12.1%) Hypochondriacal disorder (F45.2, *n* = 2, 0.6%) Somatoform autonomic dysfunction (F45.3, *n* = 56, 16.5%) Persistent somatoform pain disorder (F45.4, *n* = 168, 49.4%) Other somatoform disorders (F45.8, *n* = 12, 3.5%) Somatoform disorder, unspecified (F45.9, *n* = 3, 0.9%)	Anorexia nervosa (F50.0, *n* = 1736, 60.0%) Atypical anorexia nervosa (F50.1, *n* = 210, 7.6%) Bulimia nervosa (F50.2, *n* = 606, 22.0%) Atypical bulimia nervosa (F50.3, *n* = 97, 3.5%) Other eating disorders (F50.8, *n* = 64, 2.3%) Eating disorder, unspecified (F50.9, *n* = 41, 1.5%)

### Data analyses

*Sample characteristics*. Groups were compared regarding sex distribution with a χ^2^-test and regarding age and length of stay with univariate analyses of variance using IBM SPSS Statistics version 24.

*Internal reliability, factor structure, and measurement invariance*. Internal reliability of the SWLS was evaluated with McDonald’s ω (as has been recommended [18–21]), which was calculated with JASP version 0.11.1 (https://jasp-stats.org [22]). Confirmatory factor analysis was conducted with the structural equation modeling module of JASP, which is based on the R-package lavaan (https://lavaan.ugent.be). Diagonally Weighted Least Squares was chosen as estimation method because of the ordinal scale structure [23]. In line with previous studies [4, 5], a one-factor model was specified. Model fit was considered as good according to the recommendations by Schermelleh-Engel et al. [17]: Comparative Fit Index (CFI) ≥ 0.97, Goodness of Fit Index (GFI) ≥ 0.95, Root Mean Square Error of Approximation (RMSEA) ≤ 0.05, and Standardized Root Mean Square Residual (SRMR) ≤ 0.05. Measurement invariance across groups was tested at four levels: configural invariance (tests if the configuration of the model is the same across groups), metric invariance (tests if the factor loadings are the same across groups), scalar invariance (tests if the intercepts are the same across groups), and strict invariance (tests if the residual variances are the same across groups). There are different recommendations of how to evaluate measurement invariance but a fairly well established guideline is that model fit changes of ΔCFI ≤ 0.01 indicate invariance [24, 25]. We do not report the χ^2^-test of exact fit or χ^2^-difference tests between models because these are usually significant in large samples and, therefore, uninformative in the current sample. All analyses on internal reliability, factor structure, and measurement invariance were run separately for SWLS scores at admission and discharge.

*Life satisfaction as a function of group and time*. An analysis of variance for repeated measures was calculated using IBM SPSS Statistics version 24 with group (depressive episode vs. recurrent depressive disorder vs. phobic disorders vs. obsessive–compulsive disorder vs. trauma-related disorders vs. somatoform disorders vs. eating disorders) as between-subjects factor, time (admission vs. discharge) as within-subjects factor and SWLS scores as dependent variable.

*Changes in life satisfaction as a function of sex, age, and length of stay*. Three linear regression analyses were calculated separately with sex, age, and length of stay as independent variable with PROCESS version 3.4 (https://processmacro.org [26]). Group was entered as multicategorical moderator variable using indicator coding [27] and changes in SWLS scores (SWLS scores at discharge minus SWLS scores at admission) were entered as dependent variable.

*Depressive symptoms as a function of group and time*. An analysis of variance for repeated measures was calculated using IBM SPSS Statistics version 24 with group (depressive episode vs. recurrent depressive disorder vs. phobic disorders vs. obsessive–compulsive disorder vs. trauma-related disorders vs. somatoform disorders vs. eating disorders) as between-subjects factor, time (admission vs. discharge) as within-subjects factor and PHQ–9 scores as dependent variable.

*Associations between life satisfaction and depressive symptoms*. Pearson correlation coefficients were calculated using IBM SPSS Statistics version 24 to examine associations between SWLS and PHQ–9 scores at admission and discharge. To examine the relationship between changes in SWLS scores from admission to discharge and changes in PHQ–9 scores from admission to discharge, a repeated measures correlation was calculated with the R-package rmcorr [28].

## Results

### Sample characteristics

Groups differed in sex distribution (χ^2^_(6)_ = 1302, *p* < 0.001, ϕ = 0.367) with patients with phobic disorders having the highest percentage of males and patients with eating disorders having the lowest percentage of males (Table 1). Groups also differed in age (*F*_(6,9642)_ = 744, *p* < 0.001, η^2^_p_ = 0.316) with patients with recurrent depressive disorder having the oldest age and patients with eating disorders having the youngest age (Table 1). They also differed in length of stay (*F*_(6,9642)_ = 556, *p* < 0.001, η^2^_p_ = 0.257) with patients with somatoform disorders staying the shortest and patients with eating disorders staying the longest (Table 1).

### Internal reliability, factor structure, and measurement invariance

**Fig. 1:**
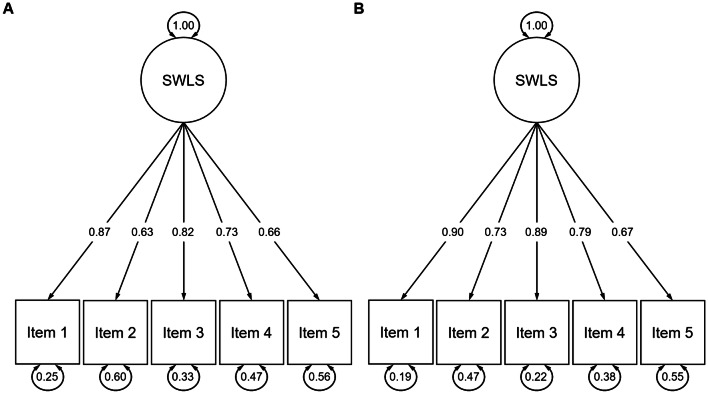
Standardized factor loadings (straight arrows) and error variances (circular arrows) of the Satisfaction With Life Scale (SWLS) items at admission (**a**) and discharge (**b**)

*Admission*. Internal reliability was good (McDonald’s ω = 0.861). The one-factor model showed a good fit (CFI = 0.997, GFI = 1.00, RMSEA = 0.043, SRMR = 0.023). Factor loadings are displayed in Fig. 1a. Examination of model fit changes indicated configural invariance (ΔCFI = 0.000 compared to the baseline model), metric invariance (ΔCFI = 0.001 compared to the configural model), scalar invariance (ΔCFI = 0.004 compared to the metric model), and strict invariance (ΔCFI = 0.002 compared to the scalar model). Accordingly, the strict invariance model still showed good model fit (CFI = 0.990, GFI = 0.998, RMSEA = 0.041, SRMR = 0.044).

*Discharge*. Internal reliability was good (McDonald’s ω = 0.897). The one-factor model showed a good fit (CFI = 0.999, GFI = 0.999, RMSEA = 0.031, SRMR = 0.019). Factor loadings are displayed in Fig. 1b. Examination of model fit changes indicated configural invariance (ΔCFI = 0.001 compared to the baseline model), metric invariance (ΔCFI = 0.002 compared to the configural model), scalar invariance (ΔCFI = 0.004 compared to the metric model), and strict invariance (ΔCFI = 0.001 compared to the scalar model). Accordingly, the strict invariance model still showed good model fit (CFI = 0.993, GFI = 0.999, RMSEA = 0.043, SRMR = 0.045).

### Life satisfaction as a function of group and time

**Fig. 2:**
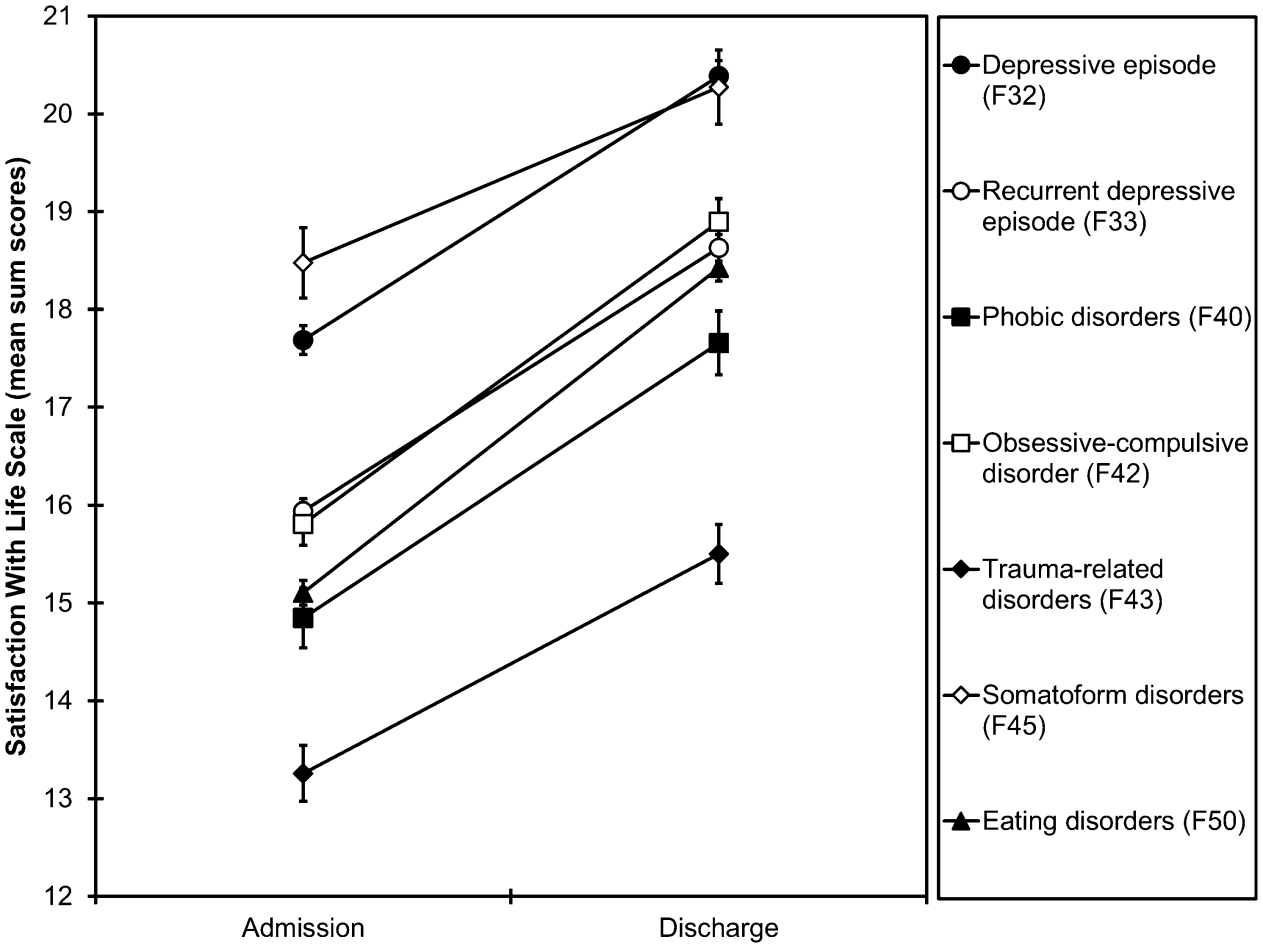
Mean sum scores of the Satisfaction With Life Scale at admission and discharge as a function of group. Error bars represent the standard error of the mean

A main effect of group (*F*_(6,9642)_ = 55.2, *p* < 0.001, η^2^_p_ = 0.033) indicated that diagnostic groups differed in life satisfaction. Patients with somatoform disorders reported the highest life satisfaction (*M* = 19.4, *SE* = 0.34) and patients with trauma-related disorders reported the lowest life satisfaction (*M* = 14.4, *SE* = 0.27; Fig. 2). A main effect of time (*F*_(1,9642)_ = 1388, *p* < 0.001, η^2^_p_ = 0.126) indicated that life satisfaction increased from admission (*M* = 15.9, *SE* = 0.09) to discharge (*M* = 18.5, *SE* = 0.10) across diagnostic groups (Fig. 2). These main effects, however, were qualified by a significant interaction of group × time (*F*_(6,9642)_ = 8.33, *p* < 0.001, η^2^_p_ = 0.005). Patients with somatoform disorders had the smallest increase in life satisfaction (*M* = 1.80, *SE* = 0.27) and patients with eating disorders had the largest increase in life satisfaction (*M* = 3.32, *SE* = 0.10). At admission, mean SWLS scores were below the neutral score of 20 [1, 4] in all groups. At discharge, mean SWLS scores were above a score of 20 only in patients with a depressive episode (*M* = 20.4, *SE* = 0.16) and patients with somatoform disorders (*M* = 20.3, *SE* = 0.38).

### Changes in life satisfaction as a function of sex, age, and length of stay

*Sex*. The interaction of sex × group was not significant (*R*^2^ change = 0.001, *F*_(6,9635)_ = 1.00, *p* = 0.425).

**Fig. 3:**
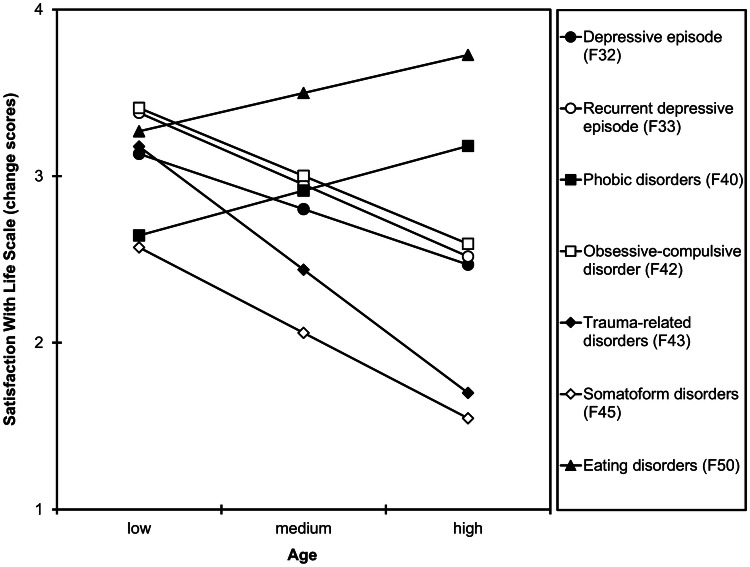
Simple slopes probing the interaction between group and age when predicting change scores of the Satisfaction With Life Scale. Higher change scores indicate larger increases in life satisfaction from admission to discharge. Low, medium, and high values for age represent 19.7 years (–1 *SD*), 36.5 years (*M*), and 53.4 years (+ 1 *SD*)

*Age*. The interaction of age × group was significant (*R*^2^ change = 0.002, *F*_(6,9635)_ = 3.32, *p* = 0.003). A younger age was significantly related to larger increases in life satisfaction in patients with a depressive episode (*b* = –0.02, *SE* = 0.01, *p* = 0.005, *r* = –0.064), recurrent depressive disorder (*b* = –0.03, *SE* = 0.01, *p* < 0.001; *r* = –0.075), and trauma-related disorders (*b* = –0.04, *SE* = 0.02, *p* = 0.007; *r* = –0.110), and marginal significantly related to larger increases in life satisfaction in patients with obsessive–compulsive disorder (*b* = –0.02, *SE* = 0.01, *p* = 0.054; *r* = –0.065) and somatoform disorders (*b* = –0.03, *SE* = 0.02, *p* = 0.084; *r* = –0.098). Age was not associated with changes in life satisfaction in patients with phobic disorders and eating disorders (both *p*s > 0.147; Fig. 3).

**Fig. 4:**
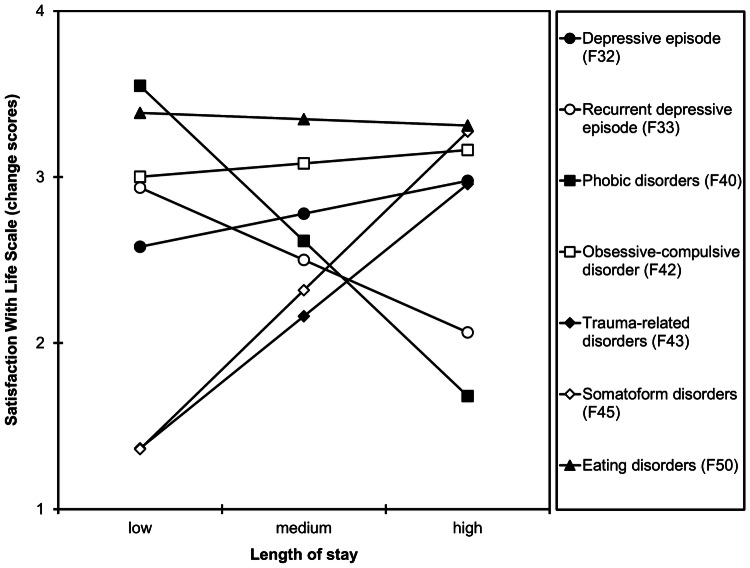
Simple slopes probing the interaction between group and length of stay when predicting change scores of the Satisfaction With Life Scale. Higher change scores indicate larger increases in life satisfaction from admission to discharge. Low, medium, and high values for length of stay represent 33.6 days (–1 *SD*), 67.9 days (*M*), and 102 days (+ 1 *SD*)

*Length of stay*. The interaction of length of stay × group was significant (*R*^2^ change = 0.002, *F*_(6,9635)_ = 3.77, *p* = 0.001). A longer stay was significantly related to larger increases in life satisfaction in patients with trauma-related disorders (*b* = 0.02, *SE* = 0.01, *p* = 0.014, *r* = 0.102) and marginal significantly related to larger increases in life satisfaction in patients with somatoform disorders (*b* = 0.03, *SE* = 0.02, *p* = 0.079, *r* = 0.100). A longer stay was significantly related to smaller increases in life satisfaction in patients with recurrent depressive disorder (*b* = –0.01, *SE* = 0.01, *p* = 0.016, *r* = –0.048) and phobic disorders (*b* = –0.03, *SE* = 0.01, *p* = 0.015, *r* = –0.118). Length of stay was not associated with changes in life satisfaction in the other groups (all *p*s > 0.228; Fig. 4).

### Depressive symptoms as a function of group and time

A main effect of group (*F*_(6,9603)_ = 61.2, *p* < 0.001, η^2^_p_ = 0.037) indicated that diagnostic groups differed in depressive symptoms. Patients with obsessive–compulsive disorder had the lowest depression scores (*M* = 9.60, *SE* = 0.17) and patients with trauma-related disorders had the highest depression scores (*M* = 13.8, *SE* = 0.22). A main effect of time (*F*_(1,9603)_ = 4074, *p* < 0.001, η^2^_p_ = 0.298) indicated that depressive symptoms decreased from admission (*M* = 13.4, *SE* = 0.08) to discharge (*M* = 8.60, *SE* = 0.08) across diagnostic groups. These main effects, however, were qualified by a significant interaction of group × time (*F*_(6,9603)_ = 33.7, *p* < 0.001, η^2^_p_ = 0.021). Patients with somatoform disorders had the smallest decrease in depressive symptoms (*M* = –3.78, *SE* = 0.27) and patients with eating disorders had the largest decrease in depressive symptoms (*M* = –6.19, *SE* = 0.11). At admission, mean PHQ–9 scores were above the cut-off score of 10 [29] in all groups. At discharge, mean PHQ–9 scores were below the cut-off score of 10 in all groups except in the patients with trauma-related disorders (*M* = 11.6, *SE* = 0.24).

### Associations between life satisfaction and depressive symptoms

At admission, SWLS and PHQ–9 scores were moderately, negatively correlated (*r* = –0.495, *p* < 0.001). At discharge, SWLS and PHQ–9 scores were highly, negatively correlated (*r* = –0.610, *p* < 0.001). The repeated measures correlation between SWLS and PHQ–9 scores within individuals across admission and discharge was *r* = –0.601 (*p* < 0.001).

## Discussion

The current study reports the largest sample of persons with mental disorders in which life satisfaction was assessed with the SWLS to date. Previous studies in this field have been limited as they mostly examined heterogeneous clinical samples (i.e., did not differentiate between different diagnostic groups), were cross-sectional (i.e., did not examined treatment changes in life satisfaction), and tested only small samples [8, 9, 12, 13]. In line with previous findings [1, 3–5, 9], the scale’s good internal reliability and one-factor structure was replicated in the current sample. Moreover, this is the first study that tested measurement invariance of the SWLS across different diagnostic groups and full measurement invariance was demonstrated. This indicates that the SWLS measures the same construct—life satisfaction—in different groups of persons with mental disorders.

### Group differences in life satisfaction

Groups significantly differed in SWLS scores. Patients with trauma-related disorders showed the lowest scores of all groups, starting at 13.3 at admission and increasing to 15.5 at discharge. In line with this, similar scores of 12.9 have been previously reported in a group of individuals with traumatic brain injury and post-traumatic stress disorder [1]. Thus, patients with trauma-related disorders are among the groups with the lowest life satisfaction, with lower scores having only been reported in the literature for male prison inmates and sex workers [1, 4]. Patients with somatoform disorders had the highest scores of 18.5 at admission. Thus, although patients with mental disorders generally report lower life satisfaction than persons without mental disorders, life satisfaction also varies considerably between different diagnostic groups.

### Treatment changes in life satisfaction

Life satisfaction increased during treatment across diagnostic groups. Patients with somatoform disorders showed the smallest increases, which may be explained by the fact that they also had the shortest stay. In line with this, a longer stay (weakly) related to larger increases in life satisfaction in this group. While each group showed a statistically significant increase in life satisfaction during inpatient treatment, only two groups increased beyond the neutral point of 20 of the SWLS [1, 4]: patients with a depressive episode (score of 20.4 at discharge) and somatoform disorders (score of 20.3 at discharge). All other groups still had mean sum scores below 20 at discharge. Thus, although life satisfaction increases during inpatient treatment, most patients are still slightly dissatisfied with their life. However, preliminary data in adolescents with anorexia nervosa from our hospital suggest that life satisfaction actually shows a further increase in the year after discharge [30]. Yet, further studies that examine long-term changes in life satisfaction after inpatient treatment in other patient groups are necessary to corroborate such an effect.

Relationships of age and length of stay with changes in life satisfaction differed across groups. A younger age was predictive of larger increases in life satisfaction, but only in some diagnostic groups such as those with a depressive episode or with recurrent depressive disorder. This is in line with the findings by Meyer et al. [12] who found a negative relationship between the duration of the disorder and life satisfaction in major depression. That is, an older age and a longer duration of the disorder seems to relate to a higher symptom severity and, similarly, to smaller changes in life satisfaction during treatment in depressive disorders. Relationships between length of stay and changes in life satisfaction even showed opposite patterns across groups: A longer stay was associated with larger increases in life satisfaction in some groups while it was inversely (or unrelated) to changes in life satisfaction in other groups. This may suggest that some patients (e.g., those with trauma-related disorders) profit from a longer treatment while in other groups (e.g., those with recurrent depressive disorder) a longer stay may reflect a therapy-resistant course.

### Differentiation between depression and life satisfaction

In line with previous findings [1, 4], depressive symptoms were negatively correlated with life satisfaction cross-sectionally. In addition, within-person correlational analyses showed that increases in life satisfaction were strongly related to decreases in depressive symptoms. Accordingly, group differences and changes in life satisfaction and depressive symptoms were largely similar. However, they were not identical. For example, the patients with obsessive–compulsive disorder had the lowest depressive symptoms although they did not report the highest life satisfaction. In addition, depressive symptoms showed a clinically significant reduction (i.e., a score lower than 10 on the PHQ–9) in almost all groups while life satisfaction showed an increase above the neutral point of the SWLS in only two groups. That is, a low depression severity is not equivalent to a high life satisfaction and a clinically significant reduction in depression severity does not necessarily imply that one is satisfied with his or her life. Together, these results corroborate that life satisfaction is related to the affective aspects of subjective well-being, but that it is also partially independent from them [1, 4, 31].

### Clinical implications

Several clinical implications can be derived from the current study. First, this study showed that inpatient treatment of mental disorders not only decreases symptoms such as depression, but also increases life satisfaction. In terms of clinical significance, however, results suggested that treatment reduced depressive symptoms more than it increased life satisfaction. This highlights the need for incorporating other therapy elements in inpatient treatment or in aftercare that are specifically designed to enhance life satisfaction (e.g., [32].). Second, the current study identified subgroups of individuals with mental disorders that may need special attention and in which such targeted interventions for improving life satisfaction might be particularly effective. For example, patients with trauma-related disorders showed the lowest life satisfaction at both admission and discharge and patients with somatoform disorders showed the smallest increase in life satisfaction from admission to discharge. Thus, these diagnostic groups may require a more intensive treatment program that focuses on life satisfaction. Third, the current study also identified moderators of treatment changes in life satisfaction. For example, in both patient groups with trauma-related and somatoform disorders, a longer stay at the hospital related to larger improvements in life satisfaction. This implies that these patients may profit from time-extended treatment.

### Limitations

The following limitations need to be considered when interpreting the current results. First, inpatients with mental disorders may not be representative of the entire population of persons with mental disorders. Specifically, inpatients usually have a higher clinical impairment and distress than outpatients or persons that do not receive treatment. Therefore, SWLS scores reported in the current study should not be treated as norm data for persons with mental disorders as these are likely lower than in persons with mental disorders who are not receiving inpatient treatment. As all patients were treated at the same hospital, site-specific effects can also not be excluded. Thus, the present findings may not be generalizable beyond the hospital, including nationally or cross-culturally. Second, in order for group sizes to be sufficiently large for performing confirmatory factor analysis, we restricted our analyses to large diagnostic groups using broad ICD–10 categories. Thus, while this study included large groups of patients with diverse mental disorders, future studies are needed that include diagnostic groups that are not part of the current sample and that differentiate between specific diagnoses within the broader diagnostic categories.

## Conclusion

In conclusion, the current study supports the excellent psychometric properties of the SWLS. Compared with SWLS scores that have been reported in the literature, persons with mental disorders in the current study reported lower life satisfaction than persons without mental disorders. Yet, the current results demonstrate that life satisfaction also varies considerably between different diagnostic groups. Finally, results show that life satisfaction increases during inpatient treatment, although at discharge most patients have rarely reached levels of life satisfaction reported in non-clinical samples.
